# Relationship (in)congruency may differently impact mental health

**DOI:** 10.1016/j.ijchp.2023.100376

**Published:** 2023-02-13

**Authors:** Katarzyna Adamczyk, Nicole Watkins, Agata Dębek, Dominika Kaczmarek, Nicola Łazarów

**Affiliations:** a Adam Mickiewicz University, Faculty of Psychology and Cognitive Science, Poznań, Poland; b Department of Psychology, Wesleyan University, USA

**Keywords:** (In)congruency, Relationship desire, Satisfaction with relationship status, Mental health

## Abstract

**Background:**

Being involved in romantic relationships has historically been related to better mental health compared to being single. However, research exploring heterogeneity within these status groups is still understudied. Our study examined the role of (in)congruency between relationship desire, dismissal, satisfaction with relationship status, and current relationship status on the mental health measured in terms of anxiety, depression, insomnia, and romantic loneliness.

**Method:**

The online questionnaire survey included 790 participants aged 18 – 40 (*M* = 26.51, *SD* = 5.60) at baseline and 421 at a 1-month follow-up. Participants represented five relationship statuses (single, casual dating, LAT relationships, cohabitation, and engagement/marriage).

**Results:**

Our results suggest that greater relationship desire and dismissal at baseline were associated with higher anxiety and depression in casual daters one month later, while greater relationship desire was linked to lower anxiety for individuals in living apart together relationships (LATs). Higher relationship dismissal in casual daters and engaged/married individuals was associated with lower insomnia. Higher satisfaction with relationship status was associated with lower depression in single individuals and lower romantic loneliness in cohabitors and engaged/married individuals.

**Conclusions:**

This study highlights that relationship (in)congruency may operate differently across various relationship status subgroups on mental health outcomes.

## Introduction

Past studies have demonstrated that involvement in social and intimate relationships has been associated with a range of health outcomes and well-being (Holt-Lunstad et al., 2017; Pietromonaco & Collins, 2017). However, more recent research has also suggested that mental health outcomes are not merely a function of marital status, or more broadly relationship status per se, but they are also a function of other factors such as relationship quality (Barr et al., 2016), satisfaction with relationship status (Lehmann et al., 2015), relationship desires and interests (Kislev, 2021; MacDonald & Park, 2022; Watkins & Beckmeyer, 2020), and the real or perceived constraints in relationships (Jamison & Beckmeyer, 2021). These recent studies also drew attention to the role of (in)congruency between people's relationship interests and their current relationship status (Beckmeyer & Cromwell, 2019). For example, in a sample of unmarried U.S. emerging adults, Beckmeyer and Cromwell (2019) found that single individuals with greater interest in relationship involvement reported greater depressive symptoms compared to single individuals who were not/were slightly interested in romantic relationships, and those who were in a relationship. Moreover, individuals in relationships experienced lower loneliness than single individuals who were highly interested in romantic relationships and single individuals who were not/or were slightly interested in relationships (Beckmeyer & Cromwell, 2019). Taken together, these studies emphasize the importance of not only relationship status, but also other factors of relationships such as intent, quality, and satisfaction with relationship status. Thus, when these concepts are mis-matched, for example, an individual who is single but really values romantic relationships and dislikes being single, then this incongruency between relationship status and relationship beliefs may impact one's mental health.

The general concept of (in)congruency has been addressed in several theories. For instance, the multiple discrepancies theory (MDT) suggests that when an individual's wants and needs are aligned (i.e., congruent), the individual is more satisfied, whereas great discrepancies between one's wants and needs (i.e., incongruency) reduces satisfaction (Michalos, 1985). Similarly, the cognitive-motivational theory of mental incongruity (TMI) explains how people behave when they experience the discrepancy between what they want and what they actually have (Dykstra, 1995). The TMI can be applied to predict the likelihood of stress which is “an incongruity accompanied by mental and physical tension.” (Dykstra, 1995, p. 322). For instance, individuals for whom their relationship status was not congruent with their relationship ideal (i.e., based on their attitudes toward relationships) experienced greater loneliness, which in turn was related to lower desire to be single, and higher desire to have a partner compared to those whose relationship status aligned with their ideal (i.e., congruent) (Dykstra, 1995).

The need to belong and desire for connection is considered innate (Baumeister & Leary, 1995). However, not all individuals draw connection from romantic partners. Therefore, one facet of (in)congruency could be between one's relationship status and their perception of relationship importance (i.e., desire and dismissal of relationships). Watkins and Beckmeyer (2020) indicated that romantic relationship desire reflects the degree to which individuals believe relationships are important and satisfying, whereas relationship dismissal reflects the extent to which one believes that romantic relationships are problematic and unimportant. This distinction between relationship desire and dismissal is consistent with findings from Park and colleagues (2021) showing that individuals may have approach and avoidance goals that act independently to shape beliefs about romantic relationships. Therefore, relationship desire and relationship dismissal appear to constitute separate constructs rather than two polars on a single continuum what implies the possibility of dialectical thinking of romantic relationships (Watkins & Beckmeyer, 2020) and both involves the perception of relationships in terms of satisfying, and an essential area of life, as well as requiring sacrifices and compromises (Park et al., 2021).

Relationship desire and dismissal were found to be significantly associated with several relationship outcomes (Watkins & Beckmeyer, 2020). For instance, higher relationship dismissal was correlated with less involvement in romantic relationships and lower relationship satisfaction, whereas higher relationship desire was related to higher happiness with romantic experiences and higher relationship satisfaction (Watkins & Beckmeyer, 2020). Finally, among single individuals, greater relationship desire was related to greater romantic relationship intent (i.e., wanting to be in a romantic relationship), whereas higher dismissal was related to less relationship intent (Watkins & Beckmeyer, 2020). Similarly, among single individuals, higher relationship desire was associated with lower satisfaction with being single and lower life satisfaction (MacDonald & Park, 2022), and a reduced desire for a relationship over time was related to lower life satisfaction (Kislev, 2021).

Another aspect of (in)congruency analyzed in the current study involved an individual's satisfaction with current relationship status. Based on work by Lehmann et al. (2015), satisfaction with relationship status means the satisfaction with the objective state of having a partner (partnered status) or not having a partner (single status) (Lehmann et al., 2015). Potential (in)congruency can arise when someone who is single is not satisfied with their singlehood status, or when someone who is in a relationship is not satisfied with being in a relationship. This type of satisfaction has been demonstrated to be related to well-being and be more predictive of life satisfaction and psychological distress than relationship status alone (Adamczyk, 2019; Lehmann et al., 2015). Moreover, satisfaction with relationship status has also been demonstrated to be negatively associated with romantic loneliness (Adamczyk, 2019).

Even though the aforementioned research gives several insights into the role of various factors for mental health outcomes as a function of marital/relationship status, it leaves the rather unattended issue of how (in)congruency between the people's current relationship status and their relationship desire and dismissal, and satisfaction with relationship is related to mental health outcomes, in particular among young adults for whom romantic relationship development constitutes one of the central developmental tasks (Rauer et al., 2013). Moreover, the role of relationship (in)congruency has not taken into account the contemporary statuses of romantic relationships which involve, among others, cohabitation and Living Apart Together relationships (LATs) and the growing number of individuals who choose single life throughout young adulthood (Mehta et al., 2020; Pepping et al., 2018).

Therefore, in the present investigation we examined the role of (in)congruency between individuals’ current relationship status and their relationship desire, dismissal, and satisfaction with relationship status measured in the first assessment (Time 1; T1) and mental health outcomes assessed after one month (Time 2; T2). Specifically, mental health outcomes were measured in terms of anxiety, depression, insomnia, and romantic loneliness. We analyzed these outcomes since past studies demonstrated associations between anxiety, depression, and sleep disturbances (see Alvaro et al., 2013 for a review), and within the domain of romantic relationships (Revenson et al., 2016). Further, we focused on the construct of loneliness because it reflects people's interpretation of their social circumstances (Cacioppo et al., 2002) (here, relationships status) which is related to negative health consequences, including greater insomnia symptoms (Benson et al., 2021; Kane et al., 2014; Selcuk et al., 2017).

To capture the contemporary diversity of romantic relationships we explored the role of (in)congruency for mental health across five distinct relationship status groups reflecting diverse levels of relationship commitment ranging from single status, casual dating relationships, LAT relationships, cohabitation, to engaged and married relationships as representing the highest level of commitment (Kamp Dush & Amato, 2005). Single status was defined as the lack of having a romantic partner or spouse (DePaulo & Morris, 2005). Casual relationships pertain to individuals who date one or more partners casually but do not initiate a committed relationship (Schindler et al., 2010), although individuals in casual dating relationships acknowledge the possibility of developing a committed relationship (Wesche et al., 2018). Living apart together relationships (LATs) involve individuals who are romantically linked but do not live in the same household (Ayuso, 2019). Cohabitation involves individuals in an intimate relationship who share the same household (Fitzpatrick et al., 2014). Engaged relationships involve couples who start to perceive the future status of their relationship in social, legal, or economic terms (Plopa et al., 2019). Further, since prior research suggests the necessity to assess whether different types of relationships are related to distinct outcomes (Wesche et al., 2018) and that treating relationships as homogonous groups (e.g., in a relationship versus single) may yield in null effects Claxton & van Dulmen (2013), we analyzed the mental health outcomes separately across relationship status groups. Finally, we employed the temporal assessment of the associations between relationship (in)congruency at T1 and mental health outcomes measured after one month because such a prospective assessment allows us to determine the links between risk factors and the development of health outcomes (Caruana et al., 2015).

We expected that high congruency (low incongruency) would be related to better mental health, (i.e., less anxiety, depression, romantic loneliness, and insomnia; Hypothesis 1a). Second, we hypothesized that low congruency (high incongruency) would be related to poorer mental health, (i.e., higher levels of anxiety, depression, romantic loneliness, and higher insomnia; Hypothesis 1b). Finally, regarding the deficit of studies pertaining to the link between relationship (in)congruency and mental health outcomes across diverse relationship statuses, we did not formulate the specific hypothesis with respect to the direction of differences between high/low congruency and mental health outcomes across relationship status groups. In turn, we posted an open research question (RQ1): How does the link between relationship (in)congruency at T1 and mental health outcomes at T2 vary across distinct relationship statuses with varying levels of commitment?

## Method

### Procedure

The research has been carried out in accordance with The Code of Ethics of the World Medical Association (Declaration of Helsinki) for experiments involving humans. The research was positively evaluated by the Ethics Committee for Research with the People as Study Participants at the Faculty of [blinded name] (Decision number: blinded number). All participants provided informed consent and consent to utilize the results in the peer-review publication.

The study was conducted between July 27, 2021 and October 5, 2021. Since the COVID-19 pandemic and imposed restrictions in face-to-face contacts in Poland, the survey was conducted as an online survey by using Google Forms. Internet-based tools represented a convenient method of rapid data collection (Menon & Muraleedharan, 2020) in light of the pandemic restrictions. Participants were recruited through advertisements posted on Facebook, and was targeted to Polish language speakers. The recruitment was performed via Facebook since it has been considered to possess the largest user base, broad coverage, and display less selection than for opt-in panels, and validate respondents’ identities (see Schneider & Harknett, 2022 for a discussion). Potential participants were asked to participate in a survey regarding the role of romantic relationships in people's lives.

Participation was voluntary and confidential and participants could end the study at any time. After completion of the first survey, participants were asked if they would participate in a follow-up survey one month later. If they agreed, they provided email addresses for future contact. The respondents completed the same measures at T1 and T2. Participants had a chance to participate in a lottery in which they could win vouchers to a Polish online store worth 25 PLN at T1 and 30 PLN at T2. On average, it took 20 - 25 minutes to answer the questions.

### Participants

Initially, 894 participants begun the study, however we excluded 104 participants (11.63%) who begun the survey but did not complete it (*n* = 35), who completed the survey more than once (*n* =7), and those who completed the survey in less than 20 minutes or more than 25 (*n* = 19), those who did not met the criterion of being young adult (i.e., between ages 18 – 40; Staudinger & Bluck, 2001) (*n* = 31), and participants who did not provide information regarding their relationship status (*n* = 12). Therefore, eligible participants at T1 comprised of 790 individuals aged 18 – 40 (*M* = 26.51, *SD* = 5.60) who identified themselves as Polish language speakers (see Table 1 for demographic information) .

**Table 1:** Sample characteristics at Time 1 and Time 2 and stratified by relationship status.

Variable	Total sample at T1	Total sample at T2	Single status	Casual dating	LATs	Cohabitation	Engagement/Marriage
N (%)	790	421	147 (18.60%)	67 (8.50%)	208 (26.30%)	169 (21.40%)	199 (25.20%)
Age, years							
Range	18 - 40	18 - 40	18 - 39	18 - 40	18 – 40	19 – 40	19 - 40
*M (SD)*	26.51 (5.60)	26.01 (5.44)	25.20 (5.51)	24.70 (5.38)	23.99 (4.97)	27.17 (4.99)	30.18 (4.81)
Gender, *n* (%)							
Male	230 (29.10%)	82 (10.40%)	46 (31.30%)	20 (29.90%)	60 (28.80%)	48 (28.40%)	56 (28.10%)
Female	536 (67.80%)	330 (41.80%)	95 (64.60%)	45 (67.20%)	138 (66.30%)	117 (69.20%)	141 (70.90%)
Other	24 (3.00%)	9 (1.10%)	6 (4.10%)	2 (3.00%)	10 (4.80%)	4 (2.40%)	2 (1.00%)
Sexual orientation							
Heterosexual	513 (64.90%)	259 (61.50%)	87 (59.20%	36 (53.70%)	124 (59.60%)	106 (62.70%)	160 (80.40%)
Lesbian/Gay	68 (8.60%)	33 (7.80%)	12 (8.20%)	9 (13.40%)	24 (11.50%)	17 (10.10%)	6 (3.00%)
Bisexual	149 (18.90%)	91 (21.60%)	25 (17.00%)	13 (19.40%)	47 (22.60%)	38 (22.50%)	26 (13.10%)
Other	28 (3.50%)	18 (2.30%)	10 (6.80%)	5 (7.50%)	6 (2.90%)	5 (3.00%)	2 (1.00%)
I do not know	32 (4.10%)	20 (4.80%)	13 (8.80%)	4 (6.00%)	7 (3.40%)	3 (1.80%)	5 (2.50%)
Place of residence, *n* (%)							
City < 200,000	328 (41.50%)	166 (39.43%)	67 (45.60%)	26 (38.80%)	110 (52.80%)	42 (24.80%)	83 (41.70%)
City > 200,000	462 (58.50%)	255 (60.57)%	80 (54.40%)	41 (61.20%)	98 (47.20%)	127 (75.20%)	116 (58.30%)
Highest education, *n* (%)							
Secondary or lower education	146 (18.50%)	71 (16.87%)	32 (21.80%)	14 (20.90%)	46 (22.10%)	27 (16.00%)	27 (13.50%)
Higher education	421 (53.30%)	216 (51.30%)	66 (44.90%)	27 (40.30%)	80 (38.50%)	98 (58.00%)	150 (75.40)
Student	223 (28.20%)	134 (31.83%)	49 (33.30%)	26 (38.80%)	82 (39.40%)	44 (26.00%)	22 (11.10%)
Singlehood length (in years); *M (SD)*	3.82 (6.93)	4.41 (7.01)	4.83 (7.71)	2.04 (4.80)	-	-	-
Relationship length (in years), *M (SD)*	4.38 (4.36)	4.37 (4.35)	-	3.75 ms (5.56)	2.53 (2.53)	3.98 (3.10)	7.29 (5.09)
Do you have a child/children?, *n* (%)							
Yes	80 (10.10%)	42 (10.00%)	0	0	6 (2.90%)	7 (4.10%)	67 (33.70%)
No	710 (89.90%)	379 (90.00%)	147 (100%)	67 (100%)	202 (97.10%)	162 (95.90%)	132 (66.30%)

*Note.* LATs = Living Apart Together relationships; T1 = Time 1; T2 = Time 2.

Among 790 participants who completed the survey at T1, 421 participants completed the survey again after a 1-month interval (attrition = 46.71%). Those who did not complete the second assessment were more likely to be older, *F*(1,788) = 7.22, *p* = .007, η^2^ =.01; male, Cramer's *V*= .24, *p* < .001; less likely to be student, Cramer's *V* = .09, *p* = .048; more likely to be casual daters and individuals in LATs (Cramer's *V* = .16, *p* < .001); and experienced higher romantic loneliness, *F*(1,788) = 4.24, *p* = .040, η^2^ =.01 at T1 compared to those who completed the survey at T2 (see Table S1 in the online supplemental materials). The effect sizes indicate that the differences between participants who remained at T1 and T2 and those participants who dropped at T2 ranged from very small and small to moderate effects, and none of these effects were large or very large.

### Measures

Relationship desire and dismissal were measured with the Polish version (Adamczyk et al., 2022) of the 6-item Brief Measure of Relationship Importance Scale (BMRI; Watkins & Beckmeyer, 2020). Two subscales were computed, including romantic desire (2-items; e.g., “Being in a romantic relationship is very important to me”) and romantic dismissal (4-items; “I prefer not being involved in a committed romantic relationship”). Participants were asked to respond to statements using a 4-point Likert-type scale ranging from 0 (*strongly disagree) to* 3 (*strongly agree*). Reliability in the original study (α = .77 for relationship desire and α = .79 for relationship dismissal; Watkins & Beckmeyer, 2020) and in the Polish study (*ω* = .71 for relationship desire and α = .82 for relationship dismissal; Adamczyk et al., 2022) was good. In the present study, the internal consistency using McDonald's omega of relationship desire subscale in the total sample was *ω* = .77 at T1 and *ω* = .76 at T2, and of relationship dismissal subscale in the total sample was *ω* = .79 at T1 and *ω* = .84 at T2.

Satisfaction with relationship status was measured with the Polish version (Adamczyk et al., 2019) of the 5-item Satisfaction with Relationship Status Scale (ReSta; Lehmann et al., 2015) (e.g., “In general, how satisfied are you with your current status?”). Participants were asked to respond to statements using a 4-point Likert-type scale ranging from 0 (*not at all)* to 3 *(to a great extent*). Reliability in the original study (Guttman's lambda λ_2_ =.93 - .94; Lehmann et al., 2015) and in the Polish study (λ_2_ =.94; Adamczyk, 2019) was good. In the present study, the internal consistency using McDonald's omega of the 5-item subscale measuring satisfaction with relationship status in the total sample was *ω* = .95 at T1 and *ω* = .96 at T2.

Depression and anxiety were measured with the Polish version (Zawislak et al., 2020) of the Depression Anxiety Stress Scale-21 (DASS-21; Henry & Crawford, 2005; Lovibond & Lovibond, 1995). DASS-21 was also used to assess stress as a covariate. The anxiety, depression, and stress subscales include 7 items for each subscale (e.g., “I felt scared without any good reason”, “I felt that I had nothing to look forward to” and “I tended to over-react to situations”, respectively). Participants were asked to respond to statements using a 4-point Likert-type scale ranging from 0 *(did not apply to me at all)* to 3 *(applied to me very much, or most of the time*). Reliability in the non-clinical sample (α = .82 for anxiety, α = .88 for depression, and α = .90 for stress; Henry & Crawford, 2005) and in the Polish study (α = .80 for anxiety, α = .86 for depression, and α = .85 for stress; Zawislak et al., 2020) was good. In the present study, the internal consistency using McDonald's omega of the anxiety subscale in the total sample was *ω* = .84 at T1 and *ω* = .85 at T2, of the depression subscale was *ω* = .91 at T1 and *ω* = .92 at T2 and stress subscale was *ω* = .87 at T1 and . *ω* = .90 at T2.

Insomnia was measured with the Polish version (Fornal-Pawłowska et al., 2011) of the Athens Insomnia Scale (AIS; Soldato et al., 2000) (e.g., “Overall quality of sleep (no matter how long you slept)”). Participants were asked to respond to statements using a 4-point Likert-type scale ranging from 0 (*satisfactory)* to 3 (*very unsatisfactory or did not sleep at all*). The AIS is an 8-item instrument assessing sleep difficulty based on the ICD-10 criteria (e.g., difficulty in falling asleep, total sleep duration, sleep quality, and sleepiness during the day; Soldato et al., 2000). Reliability in the original study (α =.75 - 90; Soldato et al., 2000) and in the Polish study (α =.74 - 90; Fornal-Pawłowska et al., 2011) was good. In the present study, the internal consistency using McDonald's omega in the total sample was *ω* = .84 at T1 and T2.

*Romantic loneliness* was measured with the Polish version (Adamczyk & DiTommaso, 2014) of a 5-item subscale of the Social and Emotional Loneliness Scale for Adults - Short Form (SELSA-S; DiTommaso et al., 2004). A sample item includes “In the last year I had an unmet need for a close romantic relationship”. Participants were asked to respond to statements using a 7-point Likert-type scale ranging from 1 (*strongly disagree*) to 7 (*strongly agree*). Reliability in the original study (α = .87 - .90; DiTommaso et al., 2004) and in the Polish study (α = .83; Adamczyk & DiTommaso, 2014) was good. In the present study, the internal consistency using McDonald's omega of the 5-item subscale measuring romantic loneliness in the total sample was *ω* = .86 at T1 and *ω* = .85 at T2.

*Relationship status* was assessed with a single item and participants were asked “Which term best describes your current relationship status?”. Participants were coded into five distinct groups: 1) Single status (“*I have no partner/life partner/husband/wife”*); 2) Casual dating relationships (“*I do not have a partner/life partner/husband/wife and I am dating”*); 3) Living Apart Together relationships (LATs) (“*I am in a stable non-marital relationship and not living with a partner”*), 4) Cohabitation (*“I am in a stable informal relationship (non-marital) and live with a partner)”*); and 5) those who were engaged or married (“*I am engaged and living with a partner; I am engaged and do not live with a partner; I am married”*).

Covariates were measured with seven items assessing sociodemographic, psychological, and relationship covariates at T1. Specifically, participants were asked to provide their age (in years), gender (0 = male, 1 = female, 2 = other), education (0 = elementary, 1 = lower secondary, 2 = basic vocational, 3 = secondary, 4 = higher, 5 = student); place of residence (0 = village, 1 = town of up to 25,000 inhabitants, 2 = town of 25,000 to 50,000 inhabitants, 4 = town of 50,000 to 200,000 inhabitants, 5 = city of 200,000 to 500,000 inhabitants); sexual orientation (1 = heterosexual, 2 = homosexual, 3 = bisexual, 4 = don't know, 5 = other), satisfaction with romantic experiences (“Overall, I am satisfied with the romantic experiences I have had.”. The item was rated on a 4-point scale (1 = strongly disagree to 4 = strongly agree); number of partners in the past (“How many romantic partners have you ever had?”. Responses ranged from 0 to more than 10). Lastly, stress was included as a covariate, as described above.

### Analytic strategy

For the main analysis, multi-group path models were estimated in *MPlus* 8.0 (Muthén & Muthén, 1998-2017). Using the structural equation modeling framework, all four outcomes of mental health were included in the same model, with five groups identified by different relationship statuses. A main effects model was estimated with relationship desire, dismissal, and relationship status satisfaction at T1, predicting each mental health outcome at T2. Further, stability paths were included for each mental health outcome (e.g., insomnia at T1 predicting insomnia and T2), as well as sociodemographic, psychological, and relationship behavior control variables. Due to the performance of the multiple comparisons, we employed the Benjamini-Hochberg adjusted *p* value and its significant was determined using a False Discovery Rate (FDR) at *p* = .05 (Benjamini & Hochberg, 1995). The adjustment of *p*-value was conducted by using FDR online calculator: https://tools.carbocation.com/FDR.

Full information maximum likelihood (FIML) was used to address missing data (Enders & Bandalos, 2001). To determine good model fit, we used fit indices consistent with the literature: Comparative fit indices (CFI) > 0.95, and root mean square error approximation (RMSEA) and standardized root mean squared (SRMR) < .05, but sufficient <.08 (Browne & Cudeck, 1992). Further, a non-significant global chi-square test of model fit is ideal; but given the large sample size, this was not considered a requirement (Little, 2013).

## Results

The descriptive statistics of major studies variables and the matrix of correlations are provided in Table S2–S4 in the online supplemental materials. For the main effects path model, the model had adequate fit, χ^2^(60) = 108.61, *p* < .001; RMSEA = .07 [.05-.09]; CFI = 0.97; SRMR = .05. See Table 2 for a concise table highlighting the results of interest, (i.e., not showing the results from the covariates) (see Tables S5–S8 in online supplemental materials for the full model results with covariates.

**Table 2:** Results from multi-group path analysis.

**Anxiety at Time 2**																								
	**Single Status (*n*=147)**					**Casual dating (*n*=67)**					**LATs (*n*=208)**					**Cohabitation (*n*=169)**					**Engagement/Marriage (*n*=199)**			
	b	*SE*	*β*	*p*		b	*SE*	*β*	*p*		b	*SE*	*β*	*p*		b	*SE*	*β*	*p*		b	*SE*	*β*	*p*
Desire	-0.01	0.12	-.01	.939		**0.63**	**0.24**	**.66**	**.006**		**-0.20**	**0.07**	**-.20**	**.008**		-0.03	0.09	-.03	.723		0.08	0.10	.09	.446
Dismissal	-0.16	0.13	-.21	.199		**1.25**	**0.38**	**.87**	**<.001**		-0.23	0.12	-.15	.059		-0.07	0.17	-.05	.672		-0.01	0.19	-.01	.963
ReStat	0.05	0.10	.07	.623		-0.20	0.19	-.20	.258		-0.03	0.09	-.03	.713		-0.17	0.12	-.15	.167		0.07	0.10	.08	.462
**Depression at Time 2**																								
	**Single Status (*n*=147)**					**Casual dating (*n*=67)**					**LATs (*n*=208)**					**Cohabitation (*n*=169)**					**Engagement/Marriage (*n*=199)**			
	b	*SE*	*β*	*p*		b	*SE*	*β*	*p*		b	*SE*	*β*	*p*		b	*SE*	*β*	*p*		b	*SE*	*β*	*p*
Desire	0.08	0.14	.09	.583		**0.49**	**0.21**	**.47**	**.020**		-0.10	0.09	-.09	.259		-0.12	0.10	-.10	.198		0.09	0.12	.08	.478
Dismissal	0.13	0.15	.13	.376		**0.74**	**0.32**	**.47**	**.007**		-0.26	0.15	-.15	.070		-0.30	0.18	-.18	.096		-0.01	0.22	-.01	.951
ReSta	**-0.25**	**0.12**	**-.28**	**.033**		-0.05	0.16	-.04	.764		-0.02	0.11	-.01	.873		-0.17	0.13	-.14	.202		-0.21	0.12	-.20	.081
**Insomnia at Time 2**																								
	**Single Status (*n*=147)**					**Casual dating (*n*=67)**					**LATs (*n*=208)**					**Cohabitation (*n*=169)**					**Engagement/Marriage (*n*=199)**			
	b	*SE*	*β*	*p*		b	*SE*	*β*	*p*		b	*SE*	*β*	*p*		b	*SE*	*β*	*p*		b	*SE*	*β*	*p*
Desire	-0.06	0.09	-.10	.482		-0.24	0.14	-.29	.073		-0.01	0.05	-.02	.778		-0.11	0.06	-.14	.050		-0.01	0.08	-.01	.941
Dismissal	0.06	0.09	.10	.487		**-0.48**	**0.22**	**-.39**	**.010**		-0.10	0.08	-.10	.203		-0.04	0.11	-.03	.745		**-0.27**	**0.14**	**-.24**	**.040**
ReSta	-0.11	0.07	-.19	.126		0.10	0.10	.12	.311		0.02	0.06	.02	.794		-0.05	0.08	-.06	.538		-0.01	0.07	-.01	.906
**Romantic Loneliness at Time 2**																								
	**Single Status (*n*=147)**					**Casual dating (*n*=67)**					**LATs (*n*=208)**					**Cohabitation (*n*=169)**					**Engagement/Marriage (*n*=199)**			
	b	*SE*	*β*	*p*		b	*SE*	*β*	*p*		b	*SE*	*β*	*p*		b	*SE*	*β*	*p*		b	*SE*	*β*	*p*
Desire	0.06	0.24	.04	.798		0.64	0.45	.43	.157		-0.15	0.12	-.09	.195		-0.02	0.08	-.01	.806		0.03	0.13	.02	.821
Dismissal	-0.19	0.25	-.11	.450		1.15	0.61	.52	.071		0.21	0.19	.08	.261		-0.20	0.15	-.09	0.208		-0.07	0.25	-.02	.790
ReSta	-0.10	0.20	-.07	.614		-0.08	0.35	-.05	.813		-0.01	0.14	.00	.970		**-0.79**	**0.13**	**-.47**	**<.001**		**-0.41**	**0.16**	**-.22**	**.013**

*Note.* ReSta = Satisfaction with relationship status; LATs = Living Apart Together relationships. T1 = Time 1; T2 = Time 2.

### Anxiety symptoms

As Table 2 displays, higher relationship desire at T1 (*β* = .66, *p* = .006) and higher relationship dismissal at T1 (*β* = .87, *p* < .001) among individuals in casual dating relationships was associated with greater anxiety. Alternatively, for individuals in LAT relationships, greater romantic desire at T1 was associated with lower anxiety symptoms at T2 (*β* = -.20, *p* = .008).

In the follow-up post-hoc analyses employing Benjamini-Hochberg adjusted *p* value and it is significant using a FDR at *p* = .05^65^ (see Table 3), the association between relationship desire and anxiety symptoms was significantly different for individuals in casual dating relationships: versus single individuals, b = -0.64, *p* = .018; versus individuals in LATs, b = -0.83, *p* = .001; versus individuals in cohabitation, b = -0.67, *p* = .010; versus engaged/married individuals, b = -0.56, *p* = .035. In further post-hoc analyses to explore the link between relationship dismissal and anxiety symptoms for individuals in LATs, the path was significantly different compared to single individuals, b = -1.41, *p* < .001; individuals in LATs, b = -1.48, *p* < .001; individuals in cohabitation, b = -1.32, *p* = .002; and engaged/married individuals, b = -1.26, *p* = .003).

**Table 3:** Post-hoc comparisons of significant paths among each relationship status group.

	RDe - A	FDR	RDi - A	FDR	RDe - D	FDR	RDi - D	FDR	ReSta - D	FDR	RDi - I	FDR	ReSta-RL	FDR
SS vs. CD	**b = -0.64 *p* = .018**	**Yes**	**b = -1.41 *p* < .001**	**Yes**	b = -0.42 *p* = .106	No	b = -0.61 *p* = .082	No	b = 0.21 *p* = .303	No	b = 0.54 *p* = .021	No	N/A	**-**
SS vs. LATs	b = 0.19 *p* = .189	No	N/A	-	N/A	-	N/A	-	b = 0.24 *p* = .146	No	N/A	-	N/A	-
SS vs. C	N/A	-	N/A	-	N/A	-	N/A	-	b = 0.08 *p* = .649	No	N/A	-	**b = 0.69 *p* = .005**	**Yes**
SS vs. EM	N/A	-	N/A	-	N/A	-	N/A	-	b = 0.04 *p* = .814	No	b = 0.33*p* = .041	No	b = 0.31*p* = .240	No
CD vs. LATs	**b = -0.83 *p* = .001**	**Yes**	**b = -1.48 *p* < .001**	**Yes**	**b = -0.60 *p* = .010**	**Yes**	**b = -1.00 *p* = .004**	**Yes**	N/A	-	b = 0.38 *p* = .104	No	N/A	-
CD vs. C	**b = -0.67 *p* = .010**	**Yes**	**b = -1.32 *p* = .002**	**Yes**	**b = -0.62 *p* = .008**	**Yes**	**b = -1.04 *p* = .004**	**Yes**	N/A	-	b = 0.44 *p* = .067	No	b = 0.71 *p* = .056	No
CD vs. EM	**b = -0.56 *p* = .035**	**Yes**	**b = -1.26 *p* = .003**	**Yes**	b = -0.41 *p* = .098	No	b = -0.75 *p* = .050	No	N/A	-	b = 0.21*p* = .413	No	b = 0.33 *p* = .393	No
LATs vs. C	b = 0.17 *p* = .153	No	N/A	-	N/A	-	N/A	-	N/A	-	N/A	-	**b = 0.78 *p* < .001**	**Yes**
LATs vs. EM	b = 0.28 *p* = .029	No	N/A	-	N/A	**-**	N/A	-	N/A	-	b = 0.17 *p* = .287	No	b = 0.40 *p* = .064	No
C vs. EM	N/A	-	N/A	-	N/A	-	N/A	-	N/A	-	b = 0.23 *p* = .174	No	b = -0.38 *p* = .070	No

*Note.* RDe = relationship desire; RDi = relationship dismissal; A = anxiety; D = depression; I – insomnia; ReSta = Satisfaction with relationship status; RL = romantic loneliness; SS = single status; CD = casual dating; C = cohabitation; LATs = Living Apart Together relationships; EM = engagement and marriage; FDR = False Discovery Rate at *p* = .05.

### Depressive symptoms

Higher relationship desire and dismissal at T1 were both associated with greater depressive symptoms at T2 (*β* = .47, *p* = .020 and *β* = .47, *p* = .007, respectively) in individuals in casual dating relationships. Upon post-hoc analyses comparing path estimates of this association (see Table 3), the link between relationship desire and depressive symptoms was significantly different between individuals in casual dating relationships and individuals in LATs (b = -0.60, *p* = .010) and between individuals in casual dating relationships and individuals in cohabitation (b = -0.62, *p* = .008). Further, the association between relationship dismissal and depressive symptoms was significantly different between individuals in casual dating relationships and individuals in LATs (b = -1.00, *p* = .004) and between individuals in casual dating relationships and individuals in cohabitation (b = -1.04, *p* = .004). Additionally, greater satisfaction with relationship status was associated with lower depressive symptoms for single individuals (*β* = -.28, *p* = .033). However, when compared to other relationship status groups in the post-hoc analyses (see Table 3), the link between status satisfaction and depressive symptoms among single individuals was not significantly different than the path estimate for any other relationship status group.

### Insomnia symptoms

As shown in Table 2, significant associations were found between relationship dismissal and insomnia symptoms for two relationship status groups. Specifically, greater relationship dismissal was associated with less insomnia symptoms in individuals in casual dating relationships (*β* = -.39, *p* = .010) and engaged/married individuals (*β* = -.24, *p* = .040). Upon post-hoc analyses comparing path estimates of this association (see Table 3), the link between relationship dismissal and insomnia was not significantly different for individuals across five distinct relationship statuses.

#### Romantic loneliness

As displayed in Table 2, greater satisfaction with relationship status at T1 was associated with lower romantic loneliness at T2 among individuals in cohabitation (*β* = -.47, *p* < .001) and engaged/married individuals (*β* = -.22, *p* = .013). In subsequent post-hoc analyses to explore the link between relationship status satisfaction and romantic loneliness, the path was significantly different between single and individuals in cohabitation (b = 0.69, *p* = .005), and individuals in LATs versus individuals in cohabitation (b = 0.78, *p* < .001; see Table 3).

## Discussion

The current investigation examined how relationship (in)congruency relates to mental health measured in terms of anxiety, depression, insomnia, and romantic loneliness after one month across five distinct relationship status groups. The study documented the complex ways that relationship (in)congruency is associated with mental health outcomes of individuals representing different relationship statuses. Our path analysis results provided mixed support for our Hypotheses 1a and 1b and the idea that five distinct relationship statuses are associated with different mental health outcomes.

Hypothesis 1a predicted that high relationship congruency at T1 would be related to better health outcomes (lower levels of negative indicators of mental health). We found partial support for this hypothesis when showing the link between (a) higher relationship desire at T1 and lower anxiety at T2 in individuals in LAT relationships, (b) greater satisfaction with relationship status at T1 and lower depression at T2 among single individuals, and (c) greater satisfaction with relationship status at T1 and lower romantic loneliness at T2 among both individuals in cohabitation and in engaged and marital relationships. H1a was therefore partially supported as we did not observe the links between high relationship dismissal among single individuals and low relationship dismissal among coupled individuals and mental health outcomes, as well as the links between relationship desire and satisfaction with relationship status were not observed for insomnia.

The first finding indicating that individuals in LAT relationships of high relationship desire reported lower anxiety after one month may be associated with the unique features of LAT relationships. To be precise, individuals in LAT relationships, in particular young adults, may not feel ready for co-residence reflecting greater commitment (Jamison & Ganong, 2011). Partners in LAT relationships may feel emotionally attached, but also may feel uncertain about their commitment in regard to maintaining the union in the future (van der Wiel et al., 2018). Further, living-apart-together enables those in the relationship to integrate and balance an individuals’ intimacy and the autonomy, flexibility and independence of being alone (van der Wiel et al., 2018). In light of these features of LAT relationships, higher romantic desire, which may reflect individuals’ emotional attachment and desire for intimacy, appears to simultaneously co-exist with individuals’ desires for autonomy, flexibility and independence. These two types of desires may be satisfied by the lack of co-residence of partners what appears to translate into lower anxiety noted in the present study.

The second finding revealed that greater satisfaction with relationship status in the first assessment was related to lower depression one month later among single individuals. This finding implies that higher congruency between single status and being satisfied with the lack of a partner translates in lower depressive symptoms. This finding is consistent with past research demonstrating that higher satisfaction with relationships status is related to lower depressive symptoms both among single and partnered individuals (Adamczyk, 2019). At the same time, higher satisfaction with single status may imply lower interest in finding a partner which goes in hand with the lack of a partner/spouse. Therefore, our finding also appears to resemble the results obtained in a study by Beckmeyer and Cromwell (2019) who demonstrated that single individuals characterized by strong interest in having a partner reported greater depressive symptoms than partnered individuals and individuals who were not or who were slightly interested in being in a relationship.

The third finding indicated that high satisfaction with relationship status in the first assessment (i.e., being satisfied with having a partner/fiancée/spouse) was related to reduced romantic loneliness after one month in individuals in cohabiting and engaged/marital unions. This beneficial function of status satisfaction for a decline in romantic loneliness is consistent with prior research showing the negative link between these variables (Adamczyk, 2019). The similar role of high satisfaction with relationship status in individuals in cohabitation and engaged/marital unions may result from the recognition that cohabitation may be a temporary stage proceeding marriage (Willoughby & Belt, 2016). Therefore, individuals in cohabiting and engaged/married relationships, which are characterized by longer mean duration than casual dating and LAT relationships, may be particularly sensitive to levels of emotional intimacy in these relationships and react to the discrepancy between desired and actual quality of social contact in the form of loneliness (De Jong Gierveld et al., 2006).

Contrary to our hypothesis (H1a), we found that higher relationship dismissal, but not romantic desire, was associated with less insomnia symptoms among those in casual dating and engaged/married relationships. This surprising mismatch between higher relationship dismissal at T1 and lower insomnia at T2 among those in casual dating and engaged/marital relationships may be that these groups are experiencing high relationship congruency. With respect to casual daters, it is possible that some of the individuals who casually date chose this type of relationships because of their high relationship dismissal, which results in lower insomnia at T2 as they experience the congruency between relationship dismissal and actual casual dating status. In turn, engaged and marital relationships represent higher levels of commitment (Kamp Dush & Amato, 2005). Therefore, such high levels of commitment reflected in the decision to marry (engagement) or formalization of the union (marriage) may be associated with the recognition that these relationships are also a source of problems (higher relationship dismissal). As a result, the acknowledgment and embracing of these features of engaged and marital relationships may translate into lower sleep problems.

In line with our Hypothesis 1b we expected that low relationship congruency would be related to worse mental health outcomes. This hypothesis was partially supported since we demonstrated that both higher relationship desire and dismissal at T1 were related to higher anxiety and depressive symptoms in individuals in casual dating relationships. This pattern of results, which requires cautious interpretation due to the low power analysis in this subgroup, suggests the dual nature of casual dating relationships. In other words, casual daters may be in the process of exploring relationship possibilities and determining whether they want to be in a relationship or not. Arguably, casual dating represents an initial stage in the formation of a relationship, and individuals at this stage of relationship development can perceive these relationships as both desirable, satisfying, and important, but that they also involve costs and sacrifices (Kefalas et al., 2011). As a result, individuals may experience both forces (i.e., being motivated to approach relationships and avoid them) (Brunstein et al., 1998) which is reflected in higher levels of anxiety and depression.

## Limitations

There are a few limitations of the current investigation to consider. First, the participants were recruited via Facebook and Polish language speakers were targeted to be enrolled in the study. Regarding that respondents in the internet survey are not selected through probability sampling (Menon & Muraleedharan, 2020), the selection of participants via Facebook might have affected the generalizability of our results. Therefore, future research would benefit from replicating our findings utilizing sample enrolled via other than internet surveys as well as including the possibility to participate the study by different modes (web/mail/telephone) to counteract those missing respondents who do not have access to the Internet or are not on Facebook (Menon & Muraleedharan, 2020).

Second, in connection with the first limitation, although the surveys conducted via Facebook recently were not found to display major biases compared to traditionally administered surveys in terms of demographics and personality traits (Kalimeri et al., 2020), it may be characterized by an over-representation of women (Whitaker et al., 2017). This Facebook user trait in connection with general gender differences in surveys (Becker, 2022), and greater difficulties in recruiting men than women in studies (Slauson-Blevins & Johnson, 2016), might have therefore resulted in an over-sampling of women in our sample. As a consequence, our findings may to greater degree reflect the associations between relationship (in)congruency and mental health outcomes among women than men.

Third, we noted a relatively high attrition rate between the first and second assessments (46.71%). Although we observed several small/moderate differences between individuals who remained in the two waves versus those who did not, the reduced sample size at T2 might negatively affect the robustness of our results. This high attrition rate might be associated with various factors, such as the online questionnaire's length (approximately 20 -25 minutes) and circumstances reducing willingness to remain in the study, which are present in face-to-face research (e.g., demand characteristics, politeness expectations, obedience to authority, conformity norms; see Hoerger, 2010). However, we attempted to alleviate bias due to attrition statistically by using full information maximum likelihood, which still utilizes data from all participants rather than list-wise deletion.

Fourth, the subgroup of individuals in casual dating relationships did not have sufficient power. As a result of the small size of the casual dating subgroup, the findings concerning this subgroup should be interpreted with caution. Fifth, in the current investigation, both assessments were conducted during the COVID-19 pandemic (July – October, 2021) and the impact of the COVID-19 pandemic and associated stress should be considered in light of recent research showing that restrictions in social contact in a response to the COVID-19 pandemic are related to negative mental health outcomes (e.g., Zhao et al., 2021). Therefore, the context of COVID-19 pandemic might affected the reported experiences in the domain of romantic relationships, in particular that both men and women were found to experience decreased relationship satisfaction across the period before and during the COVID-10 pandemic (Schmid et al., 2021).

Finally, the sample consisted of participants who were Polish language speakers, which may imply that they might have originated, or are familiar with the marital and relational context of Poland which is still hallmarked by strong adherence to traditional heterosexual marriage and has a lower prevalence and acceptance of cohabitating relationships and singlehood (Janicka & Szymczak, 2019) compared, for instance, to the United States of America. Therefore, with the context of Polish marital and relational beliefs in mind, the results of our study may differ in countries with different romantic relationship views.

## Conclusion

The current investigation advances understandings of the role of relationship (in)congruency within various relationship statuses on mental health outcomes among individuals in the period of young adulthood (i.e., ages 18 – 40). Our study contributes to existing knowledge by documenting the uniqueness of distinct types of relationship statuses and implies that relationship (in)congruency may not always translate into better or worse outcomes in all mental health domains across distinct relationship statuses. The present study provides useful implication that mental health may not be only a function of being in a relationship or being single per se but also a function of how people's relationship status is or is not congruent with their relationship attitudes reflected in terms of relationship desire and dismissal, and satisfaction with relationship status. As a result, navigation of the risks of impaired mental health requires attending to the relationship (in)congruency between what people want, and what they have.

## Conflict of interest

The authors declare no conflict of interests.

## Appendix Supplementary materials

Image, application 1

## Footnotes

Supplementary material associated with this article can be found in the online version at doi:10.1016/j.ijchp.2023.100376.
