# Supplementary material for: Relationship (in)congruency may differently impact mental health
Source: Int J Clin Health Psychol. 2023 Feb 13;23(3):100376. doi: 10.1016/j.ijchp.2023.100376 (PMC9958285; doi:10.1016/j.ijchp.2023.100376)
Supplement: Supplementary file 1 [file mmc1.docx]

**SUPPLEMENT TO** **Relationship (In)Congruency May Differently Impact** **Mental Health of Young Adults**

| **Table S1**  *Sample Characteristics of the Total Sample at T1 and T2 and as a Function of the Attrition Rate between T1 and T2* | | | | |  |
| --- | --- | --- | --- | --- | --- |
|  |  | W1-W2 Attrition Comparisons | |  |  |
| Variable |  | Participants who were  at T1and T2  (N = 421) | Participants who were  at T1 and dropped at T2  (N = 369) | Difference | Effect size   η^2^/φ/ Cramer’s *V* |
| *Demographic Variables* | | | | |  |
| Age, years |  |  |  | *p* = .007 | .01 |
| Range |  | 18 - 40 | 18 – 40 |  |  |
| *M (SD)* |  | 26.01 (5.44) | 27.08 (5.75) |  |  |
| Gender, *n* (%) |  |  |  | *p* < .001 | .24 |
| Male |  | 82 (19.48%) | 148 (40.10%) |  |  |
| Female |  | 330 (78.38%) | 206 (55.83%) |  |  |
| Other |  | 9 (2.14%) | 15 (4.07%) |  |  |
| Sexual orientation |  |  |  | *p* = .081 | .10 |
| Heterosexual |  | 259 (61.50%) | 254 (68.83%) |  |  |
| Homosexual |  | 33 (7.84%) | 35 (9.49%) |  |  |
| Bisexual |  | 91 (21.62%) | 58 (15.72%) |  |  |
| Other |  | 18 (4.28%) | 10 (2.71%) |  |  |
| I do not know |  | 20 (4.75%) | 12 (3.25%) |  |  |
| Place of residence,  *n* (%) |  |  |  |  |  |
| City < 200,000 |  | 166 (39.43%) | 162 (43.90%) | *p* = .203 | .05 |
| City > 200,000 |  | 255 (60.57)% | 207 (56.10%) |  |  |
| Highest education, *n* (%) |  |  |  | *p* = .048 | .09 |
| Secondary or lower education |  | 71 (16.86%) | 75 (20.33%) |  |  |
| Higher education |  | 216 (51.30%) | 205 (55.56%) |  |  |
| Student |  | 134 (31.83%) | 89 (24.12%) |  |  |
| Relationship status, *n* (%) |  |  |  | *p* < .001 | .16 |
| Single status |  | 71 (16.86%) | 76 (20.60%) |  |  |
| Casual dating relationships |  | 23 (5.46%) | 44 (11.92%) |  |  |
| LAT relationships |  | 128 (30.40%) | 80 (21.68%) |  |  |
| Cohabitation |  | 99 (23.52%) | 70 (18.97%) |  |  |
| Engagement and marriage |  | 100 (23.75%) | 99 (26.83%) |  |  |
| Singlehood length (in years)  *M (SD)* |  | 71 (16.86%) | 76 (20.60%) | *p* = .324 | .01 |
| Relationship duration (in years) *M (SD)* |  | 23 (5.46%) | 44 (11.92%) | *p* = .969 | .00 |
| Do you have a child/children?,  *n* (%) |  |  |  | *p* = .881 | .01 |
| Yes |  | 42 (9.98%) | 38 (10.30%) |  |  |
| No |  | 379 (90.02%) | 331 (89.70%) |  |  |
| *Psychological Variables* | | | | |  |
| Relationship desire |  | 2.16 (0.85) | 2.08 (0.87) | *p* = .212 | .00 |
| Relationship dismissal |  | 0.56 (0.67) | 0.64 (0.69) | *p* = .105 | .00 |
| Satisfaction with relationship status |  | 2.21 (0.85) | 2.13 (0.89) | *p* = .224 | .00 |
| Anxiety |  | 0.99 (0.68) | 1.01 (0.72) | *p* = .704 | .00 |
| Depression |  | 1.21 (0.87) | 1.20 (0.87) | *p* = .844 | .00 |
| Romantic loneliness |  | 2.74 (1.75) | 3.00 (1.76) | *p* = .040 | .01 |
| Insomnia |  | 0.94 (0.55) | 0.95 (0.55) | *p* = .824 | .00 |
| *Note.*  Effect size: η^2^ for continuous variables, Phi φ for dichotomous variables and Cramer’s *V* for categorical variables with two or more unique values per category. T1 = Time 1; T2 = Time 2. | | | | | |

| **Table S2**  *Descriptive Statistics of Major Variables at Time 1 and Time 2 and Stratified by Relationship Status* | | | | | | | | | | | |
| --- | --- | --- | --- | --- | --- | --- | --- | --- | --- | --- | --- |
| Variable | Total sample  at T1 | *ω* | Total sample  at T2 | *ω* | Single  status | Casual  dating | LATs | Cohabitation | Engagement/Marriage | *F* ratio | Effect size η^2^ |
| N (100%) | 790 (100%) |  | 421 (100%) |  | 147 (18.60%)  71 (16.86%) | 67 (8.50%)  23(5.46%) | 208 (26.30%)  128 (30.40%) | 169 (21.40%)  99 (23.52%) | 199 (25.20%)  100 (23.75%) | -  - | -  - |
|  | *M (SD)* |  | *M (SD)* |  | *M (SD)* | *M (SD)* | *M (SD)* | *M (SD)* | *M (SD)* |  |  |
| Relationship desire | 2.12 (0.86) | .77 | 2.14 (0.83) | .76 | 1.53 (0.97)^cde^  1.65 (1.02) ^cde^ | 1.85 (0.96)^cde^  1.89 (0.94)^e^ | 2.21 (0.80)^abcde^  2.14 (0.80)^a^ | 2.23 (0.70)^abcd^  2.26 (0.68)^a^ | 2.46 (0.65)^abc^  2.42 (0.67)^ab^ | 32.90***  10.97*** | .14  .10 |
| Relationship dismissal | 0.60 (0.68) | .79 | 0.57 (0.68) | .84 | 1.26 (0.86)^bcde^  1.23 (0.90)^cde^ | 0.84 (0.64)^acde^  0.89 (0.61) ^cde^ | 0.46 (0.51)^ab^  0.47 (0.56)^ab^ | 0.40 (0.52)^abd^  0.37 (0.44)^ab^ | 0.35 (0.43)^ab^  0.37 (0.56) ^ab^ | 66.06***  28.76*** | .25  .22 |
| Satisfaction with relationship status | 2.17 (0.87) | .95 | 2.16 (0.89) | .96 | 1.46 (0.98)^cde^  1.47 (0.98) ^cde^ | 1.51 (0.91) ^cde^  1.43 (0.99) ^cde^ | 2.42 (0.68)^ab^  2.26 (0.82) ^ab^ | 2.36 (0.65)^ab^  2.40 (0.67) ^ab^ | 2.51 (0.67) ^ab^  2.43 (0.74) ^ab^ | 63.07***  22.84*** | .24  .18 |
| Anxiety | 1.00 (0.70) | .84 | 0.84 (0.74) | .85 | 1.11 (0.72)^e^  0.93 (0.67) | 1.13 (0.69)  0.81 (0.78) | 1.03 (0.73)  0.96 (0.79)^e^ | 0.96 (0.68)  0.86 (0.82) | 0.88 (0.65)^a^  0.62 (0.59)^c^ | 3.21*  3.25* | .02  .03 |
| Depression | 1.20 (0.87) | .91 | 1.11 (0.87) | .92 | 1.48 (0.83)^bcde^  1.42 (0.87)^e^ | 1.44 (0.93)^de^  1.22 (0.98) | 1.19 (0.86)^a^  1.13 (0.91) | 1.06 (0.84)^ab^  1.07 (0.87) | 1.04 (0.85)^ab^  0.89 (0.74)^a^ | 8.12***  4.15** | .04  .04 |
| Romantic loneliness | 2.86 (1.76) | .86 | 2.78 (1.71) | .85 | 4.97 (1.41)^bcde^  4.81 (1.54)^cde^ | 4.39 (1.70)^acde^  4.35 (1.68)^cde^ | 2.35 (1.20)^ab^  2.45 (1.30) ^ab^ | 2.03 (1.16) ^ab^  2.06 (1.15) ^ab^ | 2.05 (1.28) ^ab^  2.12 (1.39) ^ab^ | 164.53***  62.47*** | .46  .38 |
| Insomnia | 0.94 (0.55) | .84 | 0.89 (0.54) | .84 | - 1. (0.56)   2. (0.58) | 0.97 (0.53)  0.89 (0.51) | 0.96 (0.57)  0.93 (0.56) | 0.90 (0.50)  0.89 (0.54) | 0.90 (0.55)  0.82 (0.51) | 1.22  0.73 | .01  .01 |
| *Note*. LATs = Living Apart Together relationships. Top raw includes the results from Time 1 (T1), whereas the bottom raw includes the results from Time (T2). *ω =* McDonald’s omega. Means with different subscripts within a row are significantly different from one another. T1 = Time 1; T2 = Time 2. Differences were significant at *** *p* < .001. ** *p* < .01. * *p* < .05. | | | | | | | | | | | |

| **Table S3**  *Additional Control Variables at T1 and T2* | | | | |
| --- | --- | --- | --- | --- |
| Measures | *M* | *SD* | *ω* |  |
| Stress T1 | 1.42 | 0.81 | .87 |  |
| Satisfaction with romantic relationships T1 | 2.69 | 1.02 | - |  |
| Number of partners T1 | 3.03 | 2.50 | - |  |
| Stress T2 | 1.39 | 0.85 | .90 |  |
| Satisfaction with romantic relationships T2 | 2.82 | 0.98 | - |  |
| Number of partners T2 | 2.32 | 1.78 | - |  |
| *Note*. T1 = Time 1; T2 = Time 2. | | | | |

| **Table S4**  *Synchronous, Auto-regressive and Cross-legged Correlations Across Measures at Time 1 and Time 2* | | | | | | | | | | | | | | | | | |
| --- | --- | --- | --- | --- | --- | --- | --- | --- | --- | --- | --- | --- | --- | --- | --- | --- | --- |
| Measures | *M* | *SD* | *ω* | 1 | 2 | 3 | 4 | 5 | 6 | 7 | 8 | 9 | 10 | 11 | 12 | 13 | 14 |
| 1. Relationship desire T1 | 2.12 | 0.86 | .77 | - | -.67*** | .12** | -.08* | -.08* | -.09** | -.29*** | **.76***** | -.66*** | .13* | -.10* | -.05 | -.11* | -.25*** |
| 2. Relationship dismissal T1 | 0.60 | 0.68 | .79 |  | - | -.22*** | .15*** | .18*** | .11** | .45*** | -.62*** | **.80***** | -.23*** | .08 | .10* | .07 | .43*** |
| 3. Satisfaction with relationship status T1 | 2.17 | 0.87 | .95 |  |  | - | -.22*** | -.45*** | -.28*** | -.68*** | .05 | -.24*** | **.79***** | -.16*** | -.41*** | -.24*** | -.69*** |
| 4. Anxiety T1 | 1.00 | 0.70 | .84 |  |  |  | - | .68*** | .49*** | .13*** | -.18*** | .18*** | -.14** | **.68***** | .48*** | .47*** | .10* |
| 5. Depression T1 | 1.20 | 0.87 | .91 |  |  |  |  | - | .48*** | .31*** | -.10* | .19*** | -.32*** | .45*** | **.69***** | .44*** | .30*** |
| 6. Insomnia T1 | 0.94 | 0.55 | .84 |  |  |  |  |  | - | .19*** | -.12* | .14** | -.27*** | .41*** | .45*** | **.74***** | .24*** |
| 7. Romantic loneliness T1 | 2.86 | 1.76 | .86 |  |  |  |  |  |  | - | -.22*** | .46*** | -.61*** | .08 | .31*** | .15** | **.86***** |
| 8. Relationship desire T2 | 2.14 | 0.83 | .76 |  |  |  |  |  |  |  | - | -.68*** | .13** | -.11* | -.09 | -.13* | -.23*** |
| 9. Relationship dismissal T2 | 0.57 | 0.68 | .84 |  |  |  |  |  |  |  |  | - | -.27*** | .09 | .16*** | .08 | .45*** |
| 10. Satisfaction with relationship status T2 | 2.20 | 0.96 | .96 |  |  |  |  |  |  |  |  |  | - | -.20*** | -.43*** | -.24*** | -.73*** |
| 11. Anxiety T2 | 0.84 | 0.74 | .85 |  |  |  |  |  |  |  |  |  |  | - | .61*** | .58*** | .15** |
| 12. Depression T2 | 1.11 | 0.87 | .92 |  |  |  |  |  |  |  |  |  |  |  | - | .57*** | .39*** |
| 13. Insomnia T2 | 0.89 | 0.54 | .84 |  |  |  |  |  |  |  |  |  |  |  |  | - | .20*** |
| 14. Romantic loneliness T2 | 2.78 | 1.71 | .85 |  |  |  |  |  |  |  |  |  |  |  |  |  | - |
| *Note*. Auto-regressive correlations are marked in bold. *ω =* McDonald’s omega. T1 = Time 1; T2 = Time 2.  *** *p* < .001. ** *p* < .01. * *p* < .05. | | | | | | | | | | | | | | | | | |

| **Table S5**  *Results for Anxiety at T2* | | | | | | | | | | | | | | | | | | | | | | | | |
| --- | --- | --- | --- | --- | --- | --- | --- | --- | --- | --- | --- | --- | --- | --- | --- | --- | --- | --- | --- | --- | --- | --- | --- | --- |
|  | **Single Status (*n*=147)** | | | |  | **Casual dating (*n*=67)** | | | |  | **LATs (*n*=208)** | | | |  | **Cohabitation (*n*=169)** | | | |  | **Engagement/Marriage (*n*=199)** | | | |
|  | b | *SE* | *β* | *p* |  | b | *SE* | *β* | *P* |  | b | *SE* | *β* | *p* |  | b | *SE* | *β* | *p* |  | b | *SE* | *β* | *p* |
| Anxiety T1 | **0.46** | **0.11** | **.49** | **<.001** |  | 0.11 | 0.27 | .08 | .704 |  | **0.58** | **0.08** | **.54** | **<.001** |  | **0.71** | **0.09** | **.61** | **<.001** |  | **0.34** | **0.10** | **.38** | **<.001** |
| Age | 0.00 | 0.02 | .01 | .973 |  | 0.06 | 0.05 | .34 | .168 |  | 0.01 | 0.01 | .05 | .561 |  | -0.01 | 0.01 | -.07 | .408 |  | 0.00 | 0.01 | .03 | .774 |
| Large city | -0.02 | 0.13 | -.02 | .867 |  | -0.23 | 0.46 | -.12 | .629 |  | -0.12 | 0.10 | -.08 | .229 |  | 0.04 | 0.14 | .02 | .796 |  | -0.01 | 0.10 | -.01 | .911 |
| Education |  |  |  |  |  |  |  |  |  |  |  |  |  |  |  |  |  |  |  |  |  |  |  |  |
| Current Student | -0.24 | 0.18 | -.17 | .179 |  | 0.37 | 0.34 | .20 | .246 |  | 0.25 | 0.15 | .16 | .092 |  | -0.30 | 0.14 | -.17 | .027 |  | 0.24 | 0.17 | .13 | .147 |
| Less than HS equivalent | -0.27 | 0.23 | -.17 | .242 |  | **1.14** | **0.45** | **.50** | **.006** |  | 0.24 | 0.17 | .13 | .160 |  | -0.18 | 0.18 | -.08 | .299 |  | 0.29 | 0.16 | .17 | .060 |
| Woman | 0.01 | 0.14 | .01 | .964 |  | 0.47 | 0.34 | .24 | .158 |  | 0.07 | 0.11 | .04 | .521 |  | 0.12 | 0.15 | .07 | .399 |  | 0.08 | 0.14 | .06 | .589 |
| Heterosexual | 0.14 | 0.14 | .10 | .300 |  | 0.46 | 0.28 | .25 | .056 |  | -0.09 | 0.10 | -.06 | .383 |  | -0.05 | 0.11 | -.03 | .630 |  | 0.03 | 0.12 | .02 | .832 |
| Stress | **0.23** | **0.10** | **.27** | **.019** |  | -0.30 | 0.30 | -.26 | .327 |  | **0.17** | **0.08** | **.19** | **.020** |  | 0.15 | 0.10 | .15 | .116 |  | 0.13 | 0.08 | .18 | .107 |
| Satisfaction w/ Rom Experiences | -0.03 | 0.07 | -.05 | .619 |  | 0.18 | 0.17 | .19 | .252 |  | 0.02 | 0.06 | .03 | .675 |  | 0.04 | 0.07 | .04 | .633 |  | -0.12 | 0.07 | -.19 | .071 |
| Number of Partners | 0.01 | 0.03 | .04 | .636 |  | 0.01 | 0.05 | .05 | .769 |  | -0.03 | 0.02 | -.08 | .227 |  | 0.03 | 0.02 | .10 | .194 |  | -0.02 | 0.03 | -.06 | .553 |
| Romantic Desire | -0.01 | 0.12 | -.01 | .939 |  | **0.63** | **0.24** | **.66** | **.006** |  | **-0.20** | **0.07** | **-.20** | **.008** |  | -0.03 | 0.09 | -.03 | .723 |  | 0.08 | 0.10 | .09 | .446 |
| Romantic Dismissal | -0.16 | 0.13 | -.21 | .199 |  | **1.25** | **0.38** | **.87** | **<.001** |  | -0.23 | 0.12 | -.15 | .059 |  | -0.07 | 0.17 | -.05 | .672 |  | -0.01 | 0.19 | -.01 | .963 |
| ReSta | 0.05 | 0.10 | .07 | .623 |  | -0.20 | 0.19 | -.20 | .258 |  | -0.03 | 0.09 | -.03 | .713 |  | -0.17 | 0.12 | -.15 | .167 |  | 0.07 | 0.10 | .08 | .462 |
| *Note*. ReSta = Satisfaction with relationship status; LATs = Living Apart Together relationships; T1 = Time 1; T2 = Time 2. | | | | | | | | | | | | | | | | | | | | | | | | |

| **Table S6**  *Results for Depression at T 2* | | | | | | | | | | | | | | | | | | | | | | | | |
| --- | --- | --- | --- | --- | --- | --- | --- | --- | --- | --- | --- | --- | --- | --- | --- | --- | --- | --- | --- | --- | --- | --- | --- | --- |
|  | **Single Status (*n*=147)** | | | |  | **Casual dating (*n*=67)** | | | |  | **LATs (*n*=208)** | | | |  | **Cohabitation (*n*=169)** | | | |  | **Engagement/Marriage (*n*=199)** | | | |
|  | b | *SE* | *β* | *p* |  | b | *SE* | *β* | *P* |  | b | *SE* | *β* | *P* |  | b | *SE* | *β* | *p* |  | b | *SE* | *β* | *p* |
| Depression T1 | **0.47** | **0.12** | **.44** | **<.001** |  | **0.77** | **0.23** | **.72** | **<.001** |  | **0.61** | **0.08** | **.59** | **<.001** |  | **0.78** | **0.07** | **.77** | **<.001** |  | **0.45** | **0.09** | **.52** | **<.001** |
| Age | -0.03 | 0.02 | -.15 | .259 |  | **0.12** | **0.04** | **.62** | **.002** |  | **0.03** | **0.02** | **.19** | **.041** |  | -0.01 | 0.02 | -.03 | .752 |  | 0.01 | 0.02 | .05 | .631 |
| Large city | -0.10 | 0.15 | -.06 | .495 |  | -0.26 | 0.47 | -.13 | .603 |  | 0.00 | 0.12 | .00 | .973 |  | -0.06 | 0.15 | -.03 | .700 |  | 0.02 | 0.11 | .01 | .882 |
| Education |  |  |  |  |  |  |  |  |  |  |  |  |  |  |  |  |  |  |  |  |  |  |  |  |
| Current Student | **-0.46** | **0.21** | -.24 | **.029** |  | 0.57 | 0.29 | .28 | .051 |  | 0.22 | 0.19 | .12 | .233 |  | -0.27 | 0.15 | -.14 | .061 |  | 0.07 | 0.20 | .03 | .709 |
| Less than HS equivalent | -0.29 | 0.27 | -.13 | .277 |  | **1.10** | **0.39** | **.45** | **.004** |  | 0.36 | 0.21 | .17 | .083 |  | 0.16 | 0.19 | .07 | .397 |  | 0.24 | 0.18 | .11 | .184 |
| Woman | **0.32** | **0.16** | .17 | **.042** |  | 0.37 | 0.32 | .18 | .271 |  | -0.09 | 0.13 | -.05 | .526 |  | 0.09 | 0.16 | .05 | .561 |  | 0.11 | 0.17 | .07 | .500 |
| Heterosexual | -0.21 | 0.16 | -.12 | .170 |  | 0.14 | 0.24 | .07 | .543 |  | 0.09 | 0.12 | .05 | .491 |  | -0.18 | 0.12 | -.10 | .136 |  | -0.06 | 0.15 | -.03 | .674 |
| Stress | 0.14 | 0.12 | .12 | .243 |  | -0.32 | 0.28 | -.26 | .249 |  | 0.12 | 0.09 | .12 | .159 |  | -0.05 | 0.10 | -.04 | .649 |  | -0.07 | 0.09 | -.08 | .448 |
| Satisfaction w/ Rom Experiences | **-0.17** | **0.08** | **-.19** | **.027** |  | -0.15 | 0.14 | -.15 | .289 |  | -0.10 | 0.07 | -.10 | .140 |  | 0.08 | 0.08 | .08 | .317 |  | -0.13 | 0.08 | -.17 | .082 |
| Number of Partners | 0.00 | 0.03 | .01 | .886 |  | 0.02 | 0.04 | .05 | .683 |  | -0.03 | 0.03 | -.06 | .406 |  | 0.02 | 0.02 | .05 | .522 |  | -0.03 | 0.03 | -.09 | .366 |
| Romantic Desire | 0.08 | 0.14 | .09 | .583 |  | **0.49** | **0.21** | **.47** | **.020** |  | -0.10 | 0.09 | -.09 | .259 |  | -0.12 | 0.10 | -.10 | .198 |  | 0.09 | 0.12 | .08 | .478 |
| Romantic Dismissal | 0.13 | 0.15 | .13 | .376 |  | **0.74** | **0.32** | **.47** | **.007** |  | -0.26 | 0.15 | -.15 | .070 |  | -0.30 | 0.18 | -.18 | .096 |  | -0.01 | 0.22 | -.01 | .951 |
| ReSta | **-0.25** | **0.12** | **-.28** | **.033** |  | -0.05 | 0.16 | -.04 | .764 |  | -0.02 | 0.11 | -.01 | .873 |  | -0.17 | 0.13 | -.14 | .202 |  | -0.21 | 0.12 | -.20 | .081 |
| *Note*. ReSta = Satisfaction with relationship status; LATs = Living Apart Together relationships; T1 = Time 1; T2 = Time 2. | | | | | | | | | | | | | | | | | | | | | | | | |

| **Table S7**  *Results for Insomnia at T2* | | | | | | | | | | | | | | | | | | | | | | | | |
| --- | --- | --- | --- | --- | --- | --- | --- | --- | --- | --- | --- | --- | --- | --- | --- | --- | --- | --- | --- | --- | --- | --- | --- | --- |
|  | **Single Status (*n*=147)** | | | |  | **Casual dating (*n*=67)** | | | |  | **LATs (*n*=208)** | | | |  | **Cohabitation (*n*=169)** | | | |  | **Engagement/Marriage (*n*=199)** | | | |
|  | b | *SE* | *β* | *p* |  | b | *SE* | *β* | *P* |  | b | *SE* | *β* | *p* |  | b | *SE* | *β* | *p* |  | b | *SE* | *β* | *p* |
| Insomnia T1 | **0.64** | **0.09** | **.64** | **<.001** |  | **1.47** | **0.19** | **.99** | **<.001** |  | **0.64** | **0.06** | **.67** | **<.001** |  | **0.71** | **0.08** | **.65** | **<.001** |  | **0.48** | **0.07** | **.54** | **<.001** |
| Age | 0.01 | 0.01 | .09 | .475 |  | **0.05** | **0.03** | **.34** | **.056** |  | 0.00 | 0.01 | .02 | .788 |  | 0.00 | 0.01 | -.03 | .724 |  | 0.01 | 0.01 | .11 | .237 |
| Large city | -0.07 | 0.09 | -.06 | .475 |  | -0.61 | 0.31 | -.38 | .020 |  | -0.06 | 0.07 | -.05 | .386 |  | 0.07 | 0.09 | .05 | .435 |  | **0.16** | **0.07** | **.17** | **.018** |
| Education |  |  |  |  |  |  |  |  |  |  |  |  |  |  |  |  |  |  |  |  |  |  |  |  |
| Current Student | 0.01 | 0.13 | .01 | .949 |  | **0.38** | **0.18** | **.24** | **.037** |  | 0.19 | 0.10 | .18 | .058 |  | -0.12 | 0.09 | -.10 | .158 |  | 0.01 | 0.12 | .01 | .907 |
| Less than HS equivalent | 0.17 | 0.16 | .13 | .279 |  | **0.73** | **0.23** | **.38** | **.003** |  | **0.26** | **0.12** | .20 | **.026** |  | -0.02 | 0.11 | -.01 | .881 |  | 0.04 | 0.12 | .03 | .713 |
| Woman | **-0.20** | **0.10** | **-.17** | **.044** |  | -0.22 | 0.19 | -.13 | .238 |  | 0.03 | 0.08 | .03 | .647 |  | 0.02 | 0.09 | .02 | .809 |  | 0.09 | 0.11 | .09 | .372 |
| Heterosexual | 0.08 | 0.09 | .07 | .374 |  | -0.21 | 0.15 | -.13 | .155 |  | -0.12 | 0.07 | -.11 | .089 |  | -0.03 | 0.07 | -.02 | .724 |  | -0.05 | 0.09 | -.04 | .567 |
| Stress | 0.10 | 0.06 | .14 | .105 |  | **-0.55** | **0.20** | **-.55** | **.001** |  | 0.02 | 0.05 | .03 | .624 |  | **0.17** | **0.06** | .24 | **.003** |  | 0.07 | 0.05 | .12 | .130 |
| Satisfaction w/ Rom Experiences | 0.01 | 0.05 | .02 | .782 |  | -0.04 | 0.09 | -.05 | .669 |  | -0.02 | 0.04 | -.03 | .600 |  | 0.00 | 0.05 | .00 | .965 |  | **-0.10** | **0.05** | **-.20** | **.035** |
| Number of Partners | -0.01 | 0.02 | -.04 | .572 |  | **-0.08** | **0.03** | **-.31** | **.007** |  | 0.02 | 0.02 | .08 | .206 |  | 0.01 | 0.01 | .04 | .595 |  | -0.01 | 0.02 | -.03 | .741 |
| Romantic Desire | -0.06 | 0.09 | -.10 | .482 |  | -0.24 | 0.14 | -.29 | .073 |  | -0.01 | 0.05 | -.02 | .778 |  | **-0.11** | **0.06** | **-.14** | **.050** |  | -0.01 | 0.08 | -.01 | .941 |
| Romantic Dismissal | 0.06 | 0.09 | .10 | .487 |  | **-0.48** | **0.22** | **-.39** | **.010** |  | -0.10 | 0.08 | -.10 | .203 |  | -0.04 | 0.11 | -.03 | .745 |  | **-0.27** | **0.14** | **-.24** | **.040** |
| ReSta | -0.11 | 0.07 | -.19 | .126 |  | 0.10 | 0.10 | .12 | .311 |  | 0.02 | 0.06 | .02 | .794 |  | -0.05 | 0.08 | -.06 | .538 |  | -0.01 | 0.07 | -.01 | .906 |
| *Note*. ReSta = Satisfaction with relationship status; LATs = Living Apart Together relationships; T1 = Time 1; T2 = Time 2. | | | | | | | | | | | | | | | | | | | | | | | | |

| **Table S8**  *Results for Romantic Loneliness at T2* | | | | | | | | | | | | | | | | | | | | | | | | |
| --- | --- | --- | --- | --- | --- | --- | --- | --- | --- | --- | --- | --- | --- | --- | --- | --- | --- | --- | --- | --- | --- | --- | --- | --- |
|  | **Single Status (*n*=147)** | | | |  | **Casual dating (*n*=67)** | | | |  | **LATs (*n*=208)** | | | |  | **Cohabitation (*n*=169)** | | | |  | **Engagement/Marriage (*n*=199)** | | | |
|  | b | *SE* | *β* | *p* |  | b | *SE* | *β* | *P* |  | b | *SE* | *β* | *p* |  | b | *SE* | *β* | *p* |  | b | *SE* | *β* | *P* |
| Loneliness T1 | **0.69** | **0.09** | **.63** | **<.001** |  | **0.53** | **0.18** | **.64** | **.002** |  | **0.60** | **0.07** | **.55** | **<.001** |  | **0.57** | **0.07** | **.57** | **<.001** |  | **0.65** | **0.08** | **.66** | **<.001** |
| Age | 0.03 | 0.04 | .10 | .456 |  | 0.01 | 0.10 | .02 | .957 |  | **0.06** | **0.02** | **.24** | **.004** |  | -0.01 | 0.01 | -.02 | .685 |  | **0.05** | **0.02** | **.20** | **.001** |
| Large city | -0.08 | 0.26 | -.03 | .748 |  | **-1.35** | **0.68** | **-.47** | **.031** |  | -0.15 | 0.16 | -.06 | .344 |  | 0.02 | 0.13 | .01 | .877 |  | 0.01 | 0.12 | .00 | .925 |
| Education |  |  |  |  |  |  |  |  |  |  |  |  |  |  |  |  |  |  |  |  |  |  |  |  |
| Current Student | 0.05 | 0.36 | .02 | .883 |  | 0.38 | 0.58 | .13 | .519 |  | 0.18 | 0.24 | .07 | .455 |  | **0.26** | **0.12** | **.10** | **.035** |  | -0.08 | 0.21 | -.02 | .715 |
| Less than HS equivalent | 0.01 | 0.46 | .00 | .992 |  | 0.99 | 0.70 | .29 | .162 |  | **0.58** | **0.27** | **.19** | **.034** |  | 0.15 | 0.16 | .05 | .346 |  | -0.10 | 0.20 | -.03 | .627 |
| Woman | -0.08 | 0.28 | -.03 | .770 |  | 0.57 | 0.49 | .19 | .243 |  | 0.14 | 0.17 | .05 | .428 |  | 0.08 | 0.13 | .03 | .541 |  | 0.04 | 0.19 | .02 | .820 |
| Heterosexual | -0.39 | 0.26 | -.13 | .130 |  | 0.51 | 0.41 | .18 | .212 |  | **0.48** | **0.16** | **.18** | **.003** |  | -0.02 | 0.10 | -.01 | .860 |  | -0.19 | 0.16 | -.06 | .240 |
| Stress | 0.29 | 0.16 | .15 | .057 |  | -0.21 | 0.43 | -.12 | .621 |  | **0.29** | **0.10** | **.19** | **.002** |  | -0.04 | 0.07 | -.03 | .573 |  | -0.04 | 0.08 | -.02 | .665 |
| Satisfaction w/ Rom Experiences | -0.19 | 0.13 | -.13 | .149 |  | 0.06 | 0.28 | .04 | .819 |  | **-0.28** | **0.09** | **-.20** | **.001** |  | 0.02 | 0.07 | .02 | .736 |  | -0.02 | 0.08 | -.01 | .842 |
| Number of Partners | -0.02 | 0.05 | -.02 | .766 |  | -0.08 | 0.07 | -.18 | .268 |  | -0.01 | 0.04 | -.01 | .863 |  | 0.01 | 0.02 | .03 | .551 |  | -0.05 | 0.03 | -.09 | .132 |
| Romantic Desire | 0.06 | 0.24 | .04 | .798 |  | 0.64 | 0.45 | .43 | .157 |  | -0.15 | 0.12 | -.09 | .195 |  | -0.02 | 0.08 | -.01 | .806 |  | 0.03 | 0.13 | .02 | .821 |
| Romantic Dismissal | -0.19 | 0.25 | -.11 | .450 |  | 1.15 | 0.61 | .52 | .071 |  | 0.21 | 0.19 | .08 | .261 |  | -0.20 | 0.15 | -.09 | 0.208 |  | -0.07 | 0.25 | -.02 | .790 |
| ReSta | -0.10 | 0.20 | -.07 | .614 |  | -0.08 | 0.35 | -.05 | .813 |  | -0.01 | 0.14 | .00 | .970 |  | **-0.79** | **0.13** | **-.47** | **<.001** |  | **-0.41** | **0.16** | **-.22** | **.013** |
| *Note*. ReSta = Satisfaction with relationship status; LATs = Living Apart Together relationships; T1 = Time 1; T2 = Time 2. | | | | | | | | | | | | | | | | | | | |  |  |  |  |  |
